# Endothelial mechanobiology

**DOI:** 10.1063/1.5129563

**Published:** 2020-02-20

**Authors:** Ming He, Marcy Martin, Traci Marin, Zhen Chen, Brendan Gongol

**Affiliations:** 1 Department of Medicine, University of California, San Diego, California 92093, USA; 2 Department of Pediatrics, Stanford University School of Medicine, Stanford, California 94305, USA; 3 Department of Health Sciences, Victor Valley College, Victorville, California 92395, USA; 4 Department of Diabetes Complications and Metabolism, Beckman Research Institute, City of Hope, California 91010, USA

## Abstract

Lining the luminal surface of the vasculature, endothelial cells (ECs) are in direct contact with and differentially respond to hemodynamic forces depending on their anatomic location. Pulsatile shear stress (PS) is defined by laminar flow and is predominantly located in straight vascular regions, while disturbed or oscillatory shear stress (OS) is localized to branch points and bifurcations. Such flow patterns have become a central focus of vascular diseases, such as atherosclerosis, because the focal distribution of endothelial dysfunction corresponds to regions exposed to OS, whereas endothelial homeostasis is maintained in regions defined by PS. Deciphering the mechanotransduction events that occur in ECs in response to differential flow patterns has required the innovation of multidisciplinary approaches in both *in vitro* and *in vivo* systems. The results from these studies have identified a multitude of shear stress-regulated molecular networks in the endothelium that are implicated in health and disease. This review outlines the significance of scientific findings generated in collaboration with Dr. Shu Chien.

## OVERVIEW OF ENDOTHELIAL MECHANOBIOLOGY

The arterial cardiovascular system is made up of a luminal endothelial layer that is essential to vascular health by responding to and relaying mechanical, paracrine, and endocrine stimulations to circulating macrophages and underlying smooth muscle cells (SMCs).^1–5^ Thus, the maintenance of endothelial health is essential for a functional vasculature and is defined by enhanced nitric oxide production and vasodilation.^6,7^ Proinflammatory stimulations, however, promote endothelial dysfunction that initiates atherosclerosis by orchestrating macrophage transendothelial migration into the vascular wall.^8^ Upon migration, macrophages polarize from an M1 to M2 phenotype and then, ultimately, into proinflammatory foam cells that act synergistically with endothelial-derived inflammatory mediators to enhance SMC proliferation.^9,10^ Ultimately, these events elicit vascular bed impairment and atherosclerotic lesion formation.^11^ Despite systemic inflammatory stimuli resulting from renal, hepatic, gastrointestinal, and pancreatic failure, atherosclerosis is a focal disease process occurring primarily at branch points and bifurcations within the arterial tree.^12^ These findings indicate that in addition to systemic stimulations, local hemodynamics elicit mechanical signaling events in endothelial cells (ECs).^13^ Thus, although each cell type plays an important role in vascular health, the mechanobiology of the endothelium is hypothesized to be an important cell type in orchestrating the focal nature of atherosclerosis.

## MECHANOTRANSDUCTION *IN VITRO* AND *IN VIVO*

**FIG. 1.**
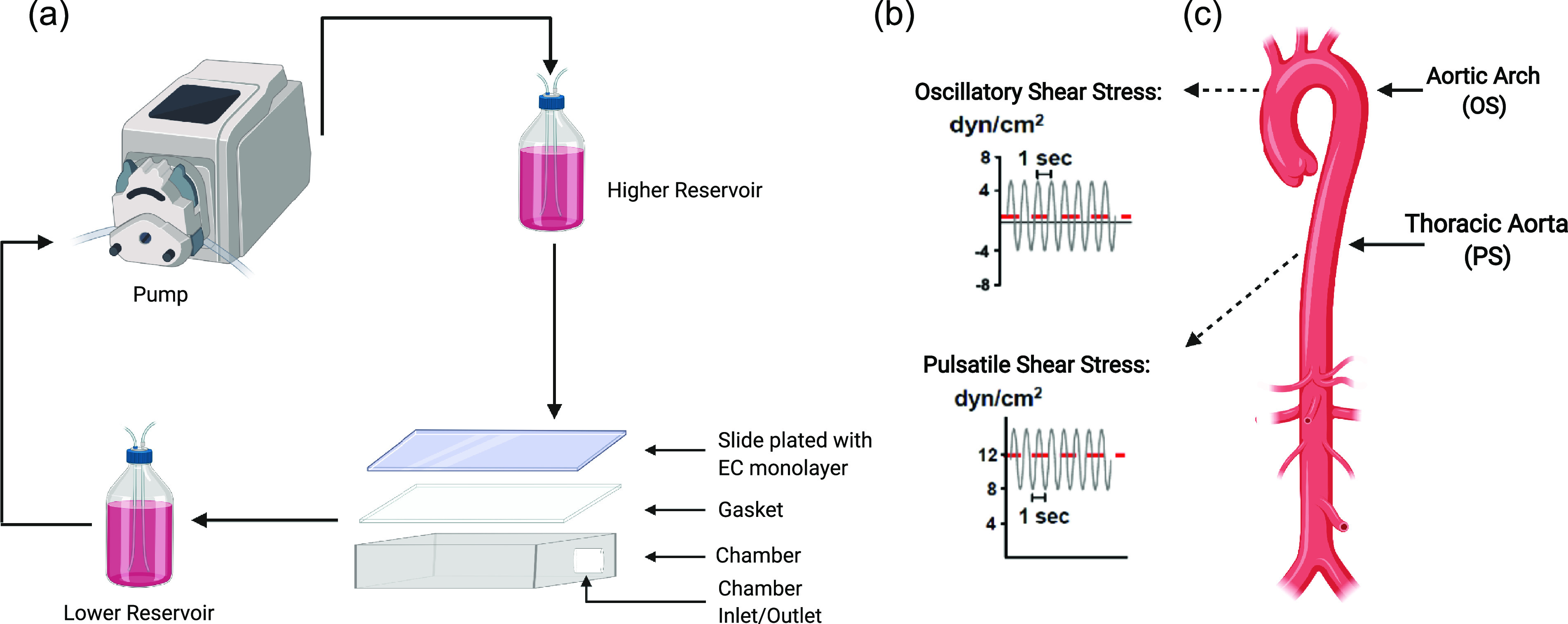
Techniques to study the effect of shear stress on the endothelium. (a) Diagram illustrating the perfusable flow system for the application of PS or OS to ECs *in vitro*. (b) The perfusion system applies shear stress, with pulsating a net directional flow rate of 12 ± 4 dynes/cm^2^, characteristic of PS (lower panel); while pulsating shear, with a minimal net directional flow rate of 0.5 ± 4 dynes/cm^2^, is characteristic of OS (upper panel). (c) Illustration of an aorta annotated with the aortic arch, a region characterized by disturbed flow patterns modeled by OS, and the thoracic aorta, a region characterized by laminar flow patterns modeled by PS.

Endothelial cells are mechanosensors that respond to physical forces caused by local blood flow patterns. Pulsatile shear stress (PS) occurs at straight regions of a vessel and is characteristically unidirectional. PS promotes EC homeostasis and vascular health. In contrast, oscillatory shear stress (OS) is associated with disturbed flow patterns that occur at vessel curvatures or bifurcations. OS induces a local inflammatory environment, promoting site-specific initiation and progression of atherosclerosis. These local shear stress patterns can be modeled *in vitro* using a parallel plate flow system [Fig. 1(a)]. In this system, ECs are plated on a glass slide as a confluent monolayer. A gasket is placed between the glass slide to form a chamber that has an inlet and outlet for the flow through culture media to create a perfusion system with regulated flow rates (e.g., 12 ± 4 dynes/cm^2^ for PS and 0.5 ± 4 dynes/cm^2^ for OS), similar to those occurring under physiological conditions [Fig. 1(b)]. Using this system, the effect of shear stress on stress fiber orientation and intracellular rheology was studied. These findings demonstrated that PS caused cytoskeletal fibers, such as actin, tubulin, and intermediate filaments, as well as the intracellular rheological parameter of creep compliance to align with the cell axis and direction of flow.^14–17^ These results led to the proposal of the novel concept that the directionality of mechanical stimulation modulates EC organizational states and hence function. To validate and complement these *in vitro* studies, *in vivo* animal experiments were conducted by comparing the thoracic aorta to the aortic arch to investigate the effects of PS and athero-protected flow vs OS and athero-prone flow, respectively [Fig. 1(c)]. Additional animal models of partial ligation (PL) include surgically ligating three branches of a common carotid artery, except the superior thyroid artery, to induce constriction which alters the flow pattern from that of athero-protective to athero-prone flow.^18–21^

*In vitro* and *in vivo* systems were used to investigate the process by which shear stress spatial-temporally regulates mechanosensors, signaling molecules, and gene regulation to influence EC phenotypes.^22^ This led to the identification of specific roles served by membrane lipids and proteins, such as receptor tyrosine kinases,^23^ junctional proteins,^24^ focal adhesion proteins,^25,26^ ion channels,^27^ G-protein coupled receptors,^28^ and integrins in flow-induced mechanotransduction.^29,30^ However, the scope of mechanotransduction-induced EC pathways extended far beyond the effect of shear stress on EC membranes and included adaptor proteins, transcription factors, receptors, kinases, junctional proteins, and adhesion molecules to name a few, some of which are summarized in Table I.^31–53^ These studies are complemented by findings from other labs.^54–62^ Of these pathways, our group collaboratively studied a portion of these mechanisms that encompassed, in part, shear regulated EC transcriptional responses.

**TABLE I.** Shear stress regulated pathways.

Class	Protein	Activation	Response
Receptors	GPCR68	PS activated	Flow mediated dilation
	PXR	PS activated	Antiinflammatory
	FLK-1	OS activated	Inflammation
	JNK	OS activated	Apoptosis
	ERK	PS activated	Increased NO bioavailability
Kinases	FAK	OS activated	Inflammation
	AMPK	PS activated	Mitochondrial function
	AKT/PI3K	PS activated	Apoptosis
	CDK	PS activated	Antiproliferation
	MAPK	PS activated	Transcriptional regulation
Junctional proteins	VE-cadherin	PS activated	Junction formation
Ion channels	Piezo1	PS activated	Cellular development
	E-selectin	PS inhibited	Inflammation
Adhesion molecules	ICAM-1	OS activated	Inflammation
	VCAM-1	OS activated	Inflammation
	MCP-1	OS activated	Monocyte adhesion
	TIFA	OS activated	Inflammation
Adaptor proteins	YAP/TAZ	OS activated	Proliferation and Inflammation
	Paxillin	OS activated	Cell orientation
	KLF2/4	PS activated	Flow mediated dilation
Transcription factors	NFκB	OS activated	Inflammation
	SMAD	OS activated	Proliferation
Deacetylases	SIRT1	PS activated	Increased NO bioavailability
Lipid metabolism	SREBP1/2	OS activated	LDL binding
Membrane dynamics	Caveolin-1	PS activated	Increased NO bioavailability
Small GTPases	Cdc42	PS activated	Cell alignment
Ubiquitination	CBL	OS activated	Neonatal hyperplasia

**FIG. 2.**
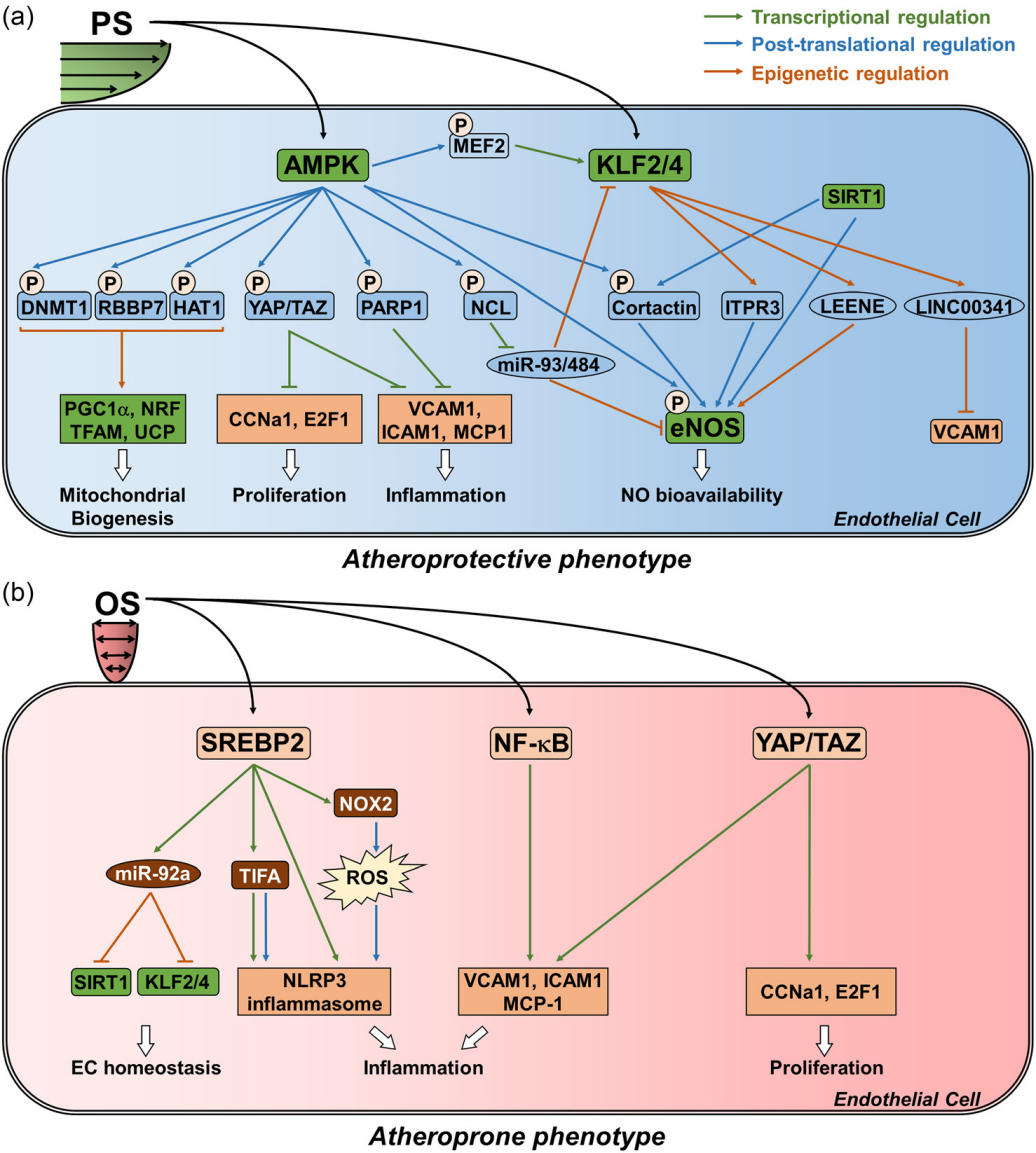
The effect of shear stress on the endothelium. (a) Network schematic for the effect of PS on ECs. PS activates AMPK, KLF2, and KLF4. AMPK is atheroprotective by phosphorylating DNMT1, RBBP7, HAT1, YAP/TAZ, PARP1, NCL, and cortactin. KLF2 exerts a similar atheroprotective effect by transcriptionally inducing ITPR3, LEENE, and LINC00341. Collectively, the activities of these targets are modulated to enhance mitochondrial biogenesis and function, increase eNOS-derived NO bioavailability, and reduce EC proliferation and inflammation. (b) Network schematic for the effect of OS on ECs. OS activates SREBP2, YAP/TAZ, and NF-κB which collectively impair EC homeostasis, and enhance EC inflammation and proliferation.

A significant pathway is the PS-induced KLF2 and KLF4 that are important transcription factors in PS induced EC function in part by transcriptionally activating endothelial nitric oxide synthase (eNOS).^31,32^ eNOS synthesizes nitric oxide (NO) that is secreted by ECs to promote vascular relaxation. Subsequent work demonstrated that AMP-activated protein kinase (AMPK) is stimulated under PS, and in turn, phosphorylates and activates eNOS at Ser-633 and Ser-1177.^63,64^ This phosphorylation status primes eNOS for deactylation by SIRT1, thereby increasing the bioavailability of NO.^46^ AMPK also increases the transcriptional expression of KLF2 via the activation of myocyte enhancement factor 2 (MEF2).^43^ This explains, in part, why AMPK activators, such as metformin and statins, exert a protective action against atherosclerosis [Fig. 2(a)].^65,66^

A hallmark of atherosclerosis is the accumulation of foam cells in the vascular wall. Under the conditions of OS, ECs have an augmented expression of monocytic chemoattractant protein 1 (MCP1).^67,68^ MCP1 recruits monocytes to the vascular wall, where they infiltrate the subendothelial space and differentiate into macrophages that scavenge oxidized lipids to form foam cells. Many other pro-inflammatory signaling pathways are induced by OS, including the NFκB pathway and its downstream target VCAM-1 [Fig. 2(b)].^17,36^ These pathways culminate in an atherogenic environment.

OS also affects EC lipid metabolism. Sterol regulatory element-binding proteins (SREBP) 1 and 2, which are key transcription factors that transactivate genes related to cholesterol biosynthesis and uptake, are upregulated under OS.^44^ Under physiological conditions, the high intracellular sterol concentration suppresses SREBP-mediated cholesterol production. Under OS, however, SREBPs undergo sustained activation.^44^ This suggests that upstream mechanosensors such as integrins may promote lipid accumulation in ECs regardless of the intracellular sterol concentration [Fig. 2(b)].^44,69^ Furthermore, SREBP2 has been linked to the EC innate immune response through the activation of the nucleotide oligomerization domain-like receptor family pyrin domain-containing protein 3 (NLRP3) inflammasome, as well as promoting the transcription of TNF-α receptor-associated factor-interacting protein with a forkhead-associated domain (TIFA), a key mediator of NLRP3 inflammasome induction [Fig. 2(b)].^51,70^ Thus, OS-activated SREBP2 enhances a vicious cycle of unresolved inflammation. In addition to these inflammatory actions, OS promotes an atherogenic phenotype by increasing EC proliferation.^71^

Yes-associated protein (YAP) and its related coactivator, PDZ-binding motif (TAZ), are key mediators of organ size and have been implicated in OS-induced EC proliferation.^50^ Experiments using the carotid artery partial ligation (PL) model that simulates OS *in vivo*,^71^ have demonstrated that YAP/TAZ is activated under disturbed flow. Furthermore, OS upregulates cell cycle regulatory genes [e.g., cyclin A1 (CNNA1) and E2F transcription factor 1 (E2F1)] and inflammatory genes [e.g., VCAM-1 and intracellular adhesion molecule 1 (ICAM1)] in a YAP-dependent fashion. Inhibition of YAP, using morpholinos, attenuated the development of atherosclerosis in the PL model [Fig. 2(b)].^50^

Taken together, these studies established that the mechanotransduction induced by flow patterns plays a major role in determining the atherogenic or atheroprotective EC phenotypes.^17,72,73^ As such, PS is atheroprotective by inhibiting EC inflammation, proliferation, and migration; while OS has the opposite effect.

## BIG DATA AND HIGH-THROUGHPUT ANALYSES IN THE INVESTIGATION OF ENDOTHELIAL MECHANOBIOLOGY

EC responses to mechanical stimulation are governed by multiple signaling events that have not been attributed to a single cellular receptor. Early investigations of these events were limited to the available genetic and molecular biology methodologies, which did not allow assessments of cellular functions at a broad level. A global view became possible with the use of new integrative techniques such as next-generation sequencing (NGS). However, these new technologies posed difficulties in their application to interpret the large datasets in *in vivo* applications and translational science. These fundamental issues were addressed by multidisciplinary approaches that included a close collaboration between experts in experimental science, medicine, engineering, and bioinformatics. These visionary perspectives were later reflected in an elegant and highly cited review written for bioengineers.^74^ These “wet-dry” lab collaborations proved to be essential to the study of EC mechanobiology by pioneering the use of new multidisciplinary techniques including fluorescence resonance energy transfer, microarray analysis, and next-generation sequencing.^75,76^ These new research tools created an unbiased view that generated novel hypotheses to study EC mechanotransduction.

Among the molecular players in EC health, AMPK has been studied as a key regulator of EC homeostasis. The application of bioinformatic approaches has identified novel AMPK targets at the proteomic level and previously unknown functions that advanced our understanding of endothelial function in atherosclerosis.^77^ For example, AMPK increases NO bioavailability by phosphorylating cortactin, which primes it for deacetylation and activation by SIRT1. AMPK also exerts anti-inflammatory effects through the phosphorylation of poly [ADP-ribose] polymerase 1 (PARP-1), which elicits cardiovascular protective effects when activated by metformin and telmisartan [Fig. 2(a)].^18,37^ At the nucleosomal level, AMPK has been proven to orchestrate the epigenetic enzymes DNA methyltransferase 1 (DNMT1), histone acetyltransferase 1 (HAT1), and retinoblastoma binding protein 7 (RBBP7) to promote the transcription of genes important for EC mitochondrial biogenesis and function [i.e., peroxisome proliferator-activated receptor gamma coactivator 1-alpha (PGC1α), nuclear respiratory factor 1 (NRF1), transcription factor A, mitochondrial (TFAM), and uncoupling protein (UCP)] [Fig. 2(a)].^78^ These studies highlight the emergence of knowledge on the epigenetic mechanisms in the shear-stress regulation of EC functions.

## microRNA (miRNA), LONG NON-CODING RNA (lncRNA), AND EPIGENETIC REGULATION OF GENE EXPRESSION IN EC MECHANOREGULATION

In addition to identifying new signaling pathways at the proteomic level, bioinformatics platforms have been developed to compile information from publicly available large datasets to facilitate the resolution of mechanisms that orchestrate non-coding RNAs, chromatin remodeling, and gene expression. As microRNAs (miRNAs) began to emerge as novel regulators in mammalian cells, NGS and bioinformatics approaches were leveraged to interrogate the role played by miRNAs in EC mechanobiology. Integrating approaches developed through bioengineering, RNA biology, and cardiovascular physiology, a number of mechanosensitive miRNAs were identified as crucial players in EC biology.^79–82^ For example, miR-92a, originally identified as a master regulator in angiogenesis,^83^ was found to be induced by OS and in turn suppressed several transcripts important to EC biology, including KLF2 and KLF4.^80,81^ Built upon these early studies, miR-92a was discovered to act as a master regulator of EC dysfunction, especially when induced by conditions such as oxidative stress, aging, and uremia [Fig. 2(b)].^84,85^ Furthermore, because many miRNA studies in ECs focused on their transcription and specific targets, there had been limited information on how they are processed in ECs in response to flow. Integrating newly available RNA-sequencing (RNA-seq) datasets with predicted AMPK targets led to the identification of a new mechanism of PS-regulated miRNA processing, through AMPK inhibition of nucleolin (NCL) binding to pre-miR-93 and pre-miR-484 [Fig. 2(a)].^86^ These studies demonstrated the essential role of miRNA regulation within ECs, and the field soon began to realize the potential of miRNAs as messengers for intercellular communication.

The growth of knowledge has led to the concepts that flow not only regulates the EC miRNA transcriptome, but also modulates the secretion of EC miRNAs and their serving as “messengers” to other vascular cells, such as the vascular smooth muscles cells.^87,88^ In fact, the miR-ome, miR-transportome, and miR-targetome are now understood as integrated components of EC regulation that orchestrates vascular cell functions giving rise to healthy or diseased phenotypes. Understanding how these multi-level players interact with each other would provide a system-view of how the vasculature as a whole responds to protective or disease-driving stimuli. The knowledge gained from elucidating miRNA-mediated EC mechanobiology led to the appreciation of how broadly ECs function in health and disease. As the field of vascular biology began to build on these developments, the impact long non-coding RNAs (lncRNA) have on EC biology surfaced.

lncRNAs have become known as crucial regulators of EC functions. Integration of transcriptome and chromatin conformation capture profiling (i.e., RNA-seq, 4C-sequencing, and Hi-C) has led to the identification of lncRNAs as important epigenetic modulators of EC responses to shear stress. For example, the PS-induced enhancer-associated lncRNA that enhances eNOS expression (LEENE or LINC00520) increases eNOS,^89^ and LINC00341 suppresses VCAM-1 expression [Fig. 2(a)].^90^ These new findings have shown that non-coding RNAs regulate gene expression not only at the post-transcriptional level, but also by scaffolding epigenetic protein regulators together to alter the chromatin structure. Thus, it became clear that RNAs function on multiple levels in EC biology, thus calling for studies to understand how ECs epigenetically respond to shear stress on the genome-wide scale.

Studies on PS-induced epigenetic regulation in ECs have identified KLF4 as a key transcription factor in shear-regulated EC homeostasis. NGS profiling using RNA-seq, H3K27ac, and H3K4me chromatin immunoprecipitation sequencing (ChIP-seq), and assay for transposase-accessible chromatin using sequencing (ATAC-seq) was conducted following shear stress stimulation to explore KLF4-mediated chromatin remodeling and the resulting gene expression. To accomplish this novel endeavor, the acquisition and analysis of these data required cross-disciplinary coordination that includes bioinformatics, molecular biology, and bioengineering. After extensive analyses, it became clear that PS- and OS-induced H3K27ac enrichment was associated with adjacent gene expression in the ECs of 18 novel PS-upregulated genes, the most significant being inositol 1,4,5-trisphosphate receptor type 3 (ITPR3). Consistent with these findings, KLF4 ATAC-seq resolved a specific locus in the promoter region of the ITPR3 gene that was essential for chromatin accessibility of KLF4 binding and transcriptional activation. Deletion of this KLF4 binding locus in ECs using clustered regularly interspaced short palindromic repeats (CRISPR)-associated protein 9 (Cas9) blunted eNOS expression and diminished NO bioavailability [Fig. 2(a)].^91^ Taken together, these studies highlight the interrelationships among the proteome, non-coding RNAs, and epigenetic regulation that orchestrate EC gene expression and intercellular communication in response to shear stress. Understanding how ECs respond to naturally existing pathophysiological flow patterns on multiple levels provides an avenue to study aberrant EC function in disease as well as potential pharmacological interventions.

## CONCLUDING PERSPECTIVES

Collectively, the discoveries emphasized in this review encompassed research conducted in collaboration with Dr. Shu Chien and how it integrates into the field of vascular biology. Combined with the work of others, these findings contributed to a paradigm shift in the field of mechanotransduction by resolving mechanisms demonstrating the active role ECs play in the pathogenesis of vascular-related disorders, such as atherosclerosis. These seminal advancements were enabled in part through the application of innovative approaches that amalgamate disciplinary boundaries from cellular and molecular levels to translational vascular medicine that pave the way for new vascular biology studies in the whole organism.

Responding to local mechanical cues that synergize with stimulations originating throughout the organism, endothelial cells orchestrate cellular responses in vascular and subvascular regions that are implicated in a number of diseases such as pulmonary hypertension, Alzheimer's disease, vascular dementia, and pancreatic cancer.^92–96^ While current systems' biology techniques are limited to studying the effect stimulations have on the endothelium, there is a growing need to understand how the endothelium is regulated in the entire organism, and also, how cells in the local vascular wall heterogenically respond to EC secreted signals. Technological advances are enhancing our ability to generate and analyze large amounts of quantitative data to explore specific biochemical aspects of cell function on the single cell level.^97^ These new approaches are complemented by developing genome editing techniques, new genetic mouse models, and microparticle techniques for the delivery of small molecules, silencing oligonucleotides, and gene editing machinery to targeted cell types.^98–100^ Additionally, new advances in data science, automation of data analytic techniques, and enhanced data storage and sharing technologies are enhancing the ability to translate conclusions drawn from rodent models into humans. These new fields erode the distinction between disciplinary boundaries and improve our ability to conduct collaborative studies that integrate diverse scientific expertise with community public health. These growing multidisciplinary, collaborative environments will continue to transform the field of endothelial mechanobiology into an era involving the generation and interpretation of an array of unbiased biological datasets that provide a systems view of EC function in the entire organism. As the scientific community explores available data and finds new innovative approaches to explore endothelial mechanobiology, this evolving field will continue to improve human health.
